# Path Analysis of the Impact of Obesity on Postoperative Outcomes in Colorectal Cancer Patients: A Population-Based Study

**DOI:** 10.3390/jcm10132904

**Published:** 2021-06-29

**Authors:** Kuan-Chih Chung, Ko-Chao Lee, Hong-Hwa Chen, Kung-Chuan Cheng, Kuen-Lin Wu, Ling-Chiao Song

**Affiliations:** 1 Department of Anesthesiology, Chang Gung Memorial Hospital-Kaohsiung Medical Center, Kaohsiung 83301, Taiwan; s21096@ms24.hinet.net; 2 Kaohsiung Medical Center, Chang Gung University College of Medicine, Kaohsiung 83301, Taiwan; honghwachen64@macsohu.cn (H.-H.C.); kungchuancheng4@macsohu.cn (K.-C.C.); kuenlinwu82@macsohu.cn (K.-L.W.); 3 Division of Colorectal Surgery, Department of Surgery, Chang Gung Memorial Hospital-Kaohsiung Medical Center, Kaohsiung 83301, Taiwan; 4 Division of Colon & Rectal Surgery, Department of Surgery, E-DA Hospital, Kaohsiung 82445, Taiwan; kulairumi@gmail.com; 5 School of Medicine, College of Medicine, I-Shou University, Kaohsiung 82445, Taiwan

**Keywords:** obesity, colorectal cancer, postoperative outcomes

## Abstract

Background: Obesity is adversely affecting perioperative outcomes; however, long-term outcomes do not appear to be affected by excess body weight (the obesity paradox). The purpose of this study is to examine the association between obesity and surgical outcomes in patients with colorectal cancer (CRC) using data from the United States National Inpatient Sample (NIS). Methods: Patients ≥20 years old diagnosed with CRC who received surgery were identified in the 2004–2014 NIS database. Patients who were obese (ICD-9-CM code: 278.0) were matched with controls (non-obese) in a 1:4 ratio for age, sex, and severity of CRC (metastasis vs. no metastasis). Linear regression and path analysis were used to compare outcomes between obese and non-obese patients. A total of 107,067 patients (53,376 males, 53,691 females) were included in the analysis, and 7.86% were obese. Results: The rates of postoperative infection, shock, bleeding, wound disruption, and digestive system complications were significantly different between the obese and non-obese groups. The obesity group had increased incidence of postoperative infection by 1.9% (**∂**P/*∂*X = 0.019), shock by 0.25% (*∂*P/*∂*X = 0.0025), postoperative bleeding by 0.5% (*∂*P/*∂*X = 0.005), wound disruption by 0.6% (*∂*P/*∂*X = 0.006), and digestive system complications by 1.35% (*∂*P/*∂*X = 0.0135). Path analysis showed that obesity group had higher in-hospital mortality through mentioned above five complications by 66.65 × 10^−5^%, length of hospital stay by 0.32 days, and total hospital charges by 2384 US dollars. Conclusions: Obesity increases the risk of postoperative complications in patients with CRC undergoing surgery. It also increased in-hospital mortality, length of hospital stay, and total hospital charges. Therefore, patients with obesity might require a higher level of preoperative interventions and complications monitoring to improve outcomes.

## 1. Introduction

Obesity is increasing worldwide, especially in developing countries, and becoming a significant health care issue [1]. In addition to the apparent associations between obesity and conditions like cardiovascular disease (CVD) and diabetes, obesity is a significant risk factor for colorectal cancer (CRC) [1,2]. Epidemiological data suggest that obesity is associated with a 30–70% increased risk of CRC [3,4]. It is not entirely clear why obesity increases the risk of CRC. However, it has been hypothesized that adipose tissue produces several hormones and proinflammatory cytokines, which may provide a microenvironment that promotes malignant changes in cells and the subsequent development of CRC [2].

Similar to obesity, the incidence of CRC is increasing worldwide despite the availability of screening tests that can detect the disease at an early stage [5,6]. It is estimated that the global burden of CRC will increase by 60% by the year 2030 [5]. Fortunately, obesity is an influential factor that can be modified and corrected [7].

Obesity is known to affect perioperative outcomes adversely; however, long-term outcomes do not appear to be affected by excess body weight (the obesity paradox) [8]. However, little is known about the effect of obesity on the outcomes of surgery for CRC. Some study has suggested that CRC patients who are morbidly obese and undergo surgery have increased surgical complications and mortality [9]. However, other reports have indicated inconsistent findings [10].

Given the association between obesity and risk of CRC and the increasing incidence of both worldwide, the purpose of this study was to use data from a nationwide database containing information of on inpatient admissions to examine the association between obesity and surgical outcomes in patients with CRC.

## 2. Materials and Methods

### 2.1. Data Source

Data from 2004 to 2014 were extracted from the United States National Nationwide Inpatient Sample (NIS) database (https://www.hcup-us.ahrq.gov/nisoverview.jsp, accessed date 20 June 2020). The National Inpatient Sample is a United States database that is publicly available and includes information regarding non-rehabilitation and community hospital inpatient stays. It was developed for the United States Healthcare Cost and Utilization Project (HCUP). The NIS contains data from approximately 7 million hospital stays each year, and when weighted, it is estimated to cover more than 35 million hospitalizations nationally every year. Data in the NIS are sampled from State Inpatient Databases, which include all inpatient data that are currently contributed to the HCUP. The NIS was developed to provide information on hospital utilization, charges, and quality of care in the United States. Information about the NIS is available at https://www.hcup-us.ahrq.gov/nisoverview.jsp (accessed date 20 June 2020). The data contained in the NIS are de-identified, and the Institutional Review Board of Johns Hopkins Medical Institutions deemed that the study using NIS data is exempt from ethical review.

### 2.2. Study Population

**Figure 1:**
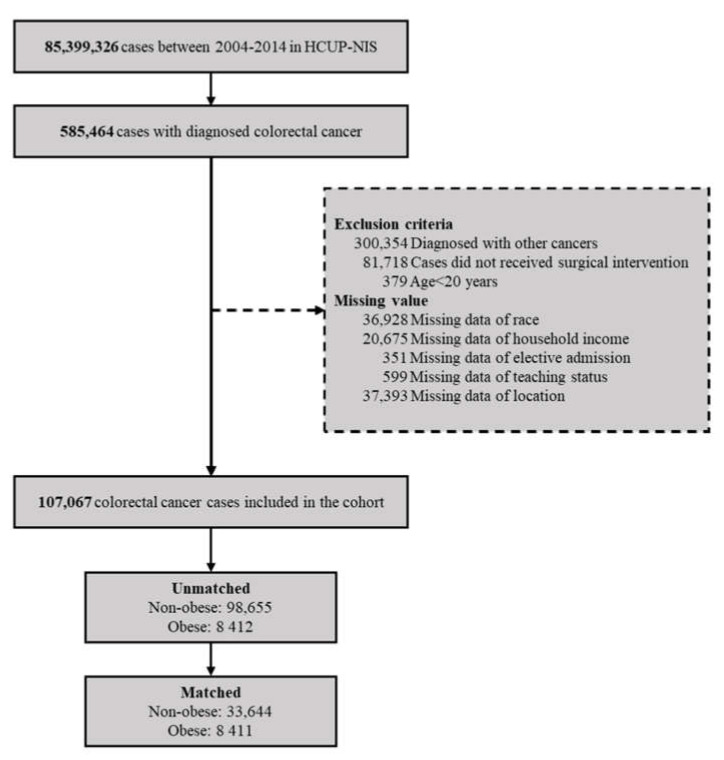
Flow diagram of the patient selection process.

Data of patients ≥20 years old diagnosed with CRC (ICD-9-CM code: 153, 154) who received surgical interventions from 2004 to 2014 were extracted from the HCUP-NIS database. Patients who also had other cancers (ICD-9-CM code: 140–239) were excluded. Surgical interventions included open and subtotal colectomy (ICD-9 procedure code 45.7), pull-through resection of the rectum (procedure code 48.40, 48.41, 48.43, 48.49), abdominoperineal resection of the rectum/complete proctectomy (procedure code 48.50, 48.52, 48.59), and other resections of the rectum (partial proctectomy, rectosigmoidectomy; ICD-9 procedure code 48.6). The ICD-9 codes of the surgical intervention used in this study are summarized in Supplementary Table S1. The patient selection process is summarized in Figure 1. Patients who were obese (ICD-9-CM code: 278.0) were matched with controls (non-obese) in a 1:4 ratio for age, sex, and severity of CRC (metastasis vs. no metastasis).

### 2.3. Study Variables

#### 2.3.1. Definition of Obesity

According to the World Health Organization, obesity is defined as abnormal or excessive fat accumulation, and a person is considered obese if her/his body mass index (BMI) is ≥30. However, the standards may vary with age, sex, genetic, or cultural background. Based on the database contents, this study uses ICD-9-CM code (278.0) to distinguish whether patients are obese.

#### 2.3.2. Dependent Variables

The primary endpoint of the study was the incidence of postoperative complications. Postoperative complications were defined by the ICD-9-CM codes, containing (1) postoperative infection (ICD-9-CM code: 998.5) including infected postoperative seroma, postoperative abscess in intra-abdominal/stitch/wound, or septicemia; (2) postoperative shock (ICD-9-CM code: 998.0): collapse during or resulting from a surgical procedure, including endotoxic/hypovolemic/septic shock; (3) postoperative bleeding (ICD-9-CM code: 998.1): hemorrhage or hematoma or seroma complicating a procedure; (4) wound disruption (ICD-9-CM = 998.3); (5) non-healing surgical wound (ICD-9-CM code: 998.83); (6) nervous system complications (ICD-9-CM code: 997.0) such as anoxic brain damage, cerebral hypoxia, or iatrogenic cerebrovascular infarction or hemorrhage/postoperative stroke; (7) cardiac arrest/heart failure (ICD-9-CM code: 997.1); (8) phlebitis/thrombophlebitis (ICD-9-CM code: 997.2); (9) respiratory complications (ICD-9-CM code: 997.3): including ventilator associated pneumonia, pneumonia (aspiration) resulting from a procedure; (10) digestive system complications (ICD-9-CM code: 997.4): complications of intestinal anastomosis and bypass, hepatic failure/hepatorenal syndrome/intestinal obstruction specified as due to a procedure; (11) urinary system complications (ICD-9-CM code: 997.5): complications of external stoma/internal anastomosis and bypass of urinary tract, oliguria or anuria/acute renal failure or insufficiency/tubular necrosis specified as due to procedure; (12) vascular complications (ICD-9-CM code: 997.7): vascular complications of mesenteric artery/renal artery/other artery following a procedure; and (13) unspecified complications of procedure (ICD-9-CM code: 998.9). The ICD-9 codes of the primary endpoint used in this study are summarized in Supplementary Table S1.

The secondary endpoints were in-hospital mortality, length of stay (days) in the hospital, and total hospital charges (in US dollars).

### 2.4. Independent Variables

#### 2.4.1. Demographic Variables

Demographic variables studied included age (20–44, 45–59, 60–74, 75+ years old), sex (male, female), race (categorized as White, Black, Hispanic, Asian or Pacific Islander, Other), type of admission (categorized as elective admission, non-elective admission), and household income. Household income was classified as quartiles of the residents’ estimated median household income in the patient’s ZIP Code. The quartiles were identified by values of 1 to 4, indicating the poorest to wealthiest populations.

#### 2.4.2. The Severity of CRC and Comorbidities

The severity of CRC was classified based on the presence of metastasis (metastasis vs. no metastasis). Comorbidities examined in the analysis included alcohol abuse, diabetes, liver disease, and data obtained based on the Agency for Healthcare Research and Quality (AHRQ) comorbidities measures, NIS Severity File Data. Complete documentation and explanations of severity measures and comorbidity measures is available on the HCUP Website (https://www.hcup-us.ahrq.gov/db/nation/nis/nisdde.jsp, accessed date 20 June 2020).

#### 2.4.3. Hospital Characteristics

Hospital characteristics examined included the number of beds (categorized as small, median, large), location (rural vs. urban), and teaching hospital or not. These data were obtained from the NIS Hospital File Data.

#### 2.4.4. Statistical Analysis

Categorical variables were presented as counts and percentages, and continuous variables as means ± standard deviation (SD). Demographic and disease characteristics, comorbidities, and clinical outcomes were compared between the obese and non-obese groups in unmatched and matched cohorts using Pearson’s chi-square test or t-test for categorical variables or continuous variables. In addition, complications significantly (*p* < 0.05) related to obesity in the multivariate logistic regression analysis were selected and followed by running path analysis in order to identify the determinants of obesity and outcomes (in-hospital mortality, length of hospital stay, and total fees) and their correlations. *∂*P/*∂*X represents the marginal probability change when the independent variable (X) changes one unit; it equals ß × P × (1 − P), where P is the probability of complications that occurred.

In this article, the term “effect” was used in two senses: First, relative effects involve ratios of these measures like Odds Ratio (OR), which are also the most popular measuring effects in most epidemiology studies. Second, absolute effects are differences in disease occurrence between two groups of people who differ concerning a causal characteristic, which is generally referred to as an “exposure”. Considering the multiplicativity for each path coefficient, the absolute marginal effect (or simply marginal effect) was chosen to measure the “effect” in this study. Otherwise, the multiplicativity of Odds Ratio (OR) would be highly complex from the mathematical perspective. In all analyses, a 2-sided P of < 0.05 was considered statistically significant. Statistical analyses were performed using the SAS statistical software package, version 9.4 (SAS Institute Inc., Cary, NC, USA).

## 3. Results

### 3.1. Baseline Data

A total of 107,067 patients ≥ 20 years old (53,376 males, 53,691 females) who met the inclusion criteria were identified in the NIS database and included in the analysis. Of the total patients, 7.86% were obese. Characteristics of the patients, categorized as obese or not obese, are summarized in Table 1. The distributions of race, household income, type of admission, diabetes, liver disease, hospital location, and hospital teaching status were significantly different between the obese and non-obese groups.

The differences in outcomes according to obesity status are summarized in Table 2. In the unmatched population, in-hospital mortality, postoperative infection, postoperative shock, wound disruption, and total hospital charges significantly differed between the obese and non-obese groups (all, *p* < 0.05). The same findings were noted in the matched population; however, postoperative bleeding, digestive system complications, and length of hospital stay were also significantly different between the obese and non-obese groups (all, *p* < 0.05).

### 3.2. The Effect of Obesity on Postoperative Complications

The effects of obesity on the five postoperative complications analyzed by multivariable logistic regression are summarized in Table 3. After adjustment for significant confounding variables, obesity increased the incidence of postoperative infection by 1.9% (*∂*P/*∂*X = 0.019), shock by 0.25% (*∂*P/*∂*X = 0.0025), postoperative bleeding by 0.5% (*∂*P/*∂*X = 0.005), wound disruption by 0.6% (*∂*P/*∂*X = 0.006), and digestive system complications by 1.35% (*∂*P/*∂*X = 0.0135).

### 3.3. The Effects of Postoperative Complications on Outcomes

The marginal effects of postoperative complications on in-hospital mortality are summarized in Table 4. Postoperative infection increased the in-hospital mortality by 1.76%, shock increased in-hospital mortality by 5.21%, bleeding increased in-hospital mortality by 2.21%, wound disruption increased in-hospital mortality by 1.32%, and digestive system complications increased in-hospital mortality by 0.46%.

As shown in Table 5, postoperative infection, shock, bleeding, wound disruption, and digestive system complications increased the hospital length of stay by 8.42, 8.68, 3.15, 11.09, and 3.94 days, respectively, and increased the total hospital charges by 62,169, 85,247, 30,770, 86,760, and 23,378 US dollars, respectively.

### 3.4. The Pathways of Obesity Affect Outcomes via the Complications

**Figure 2:**
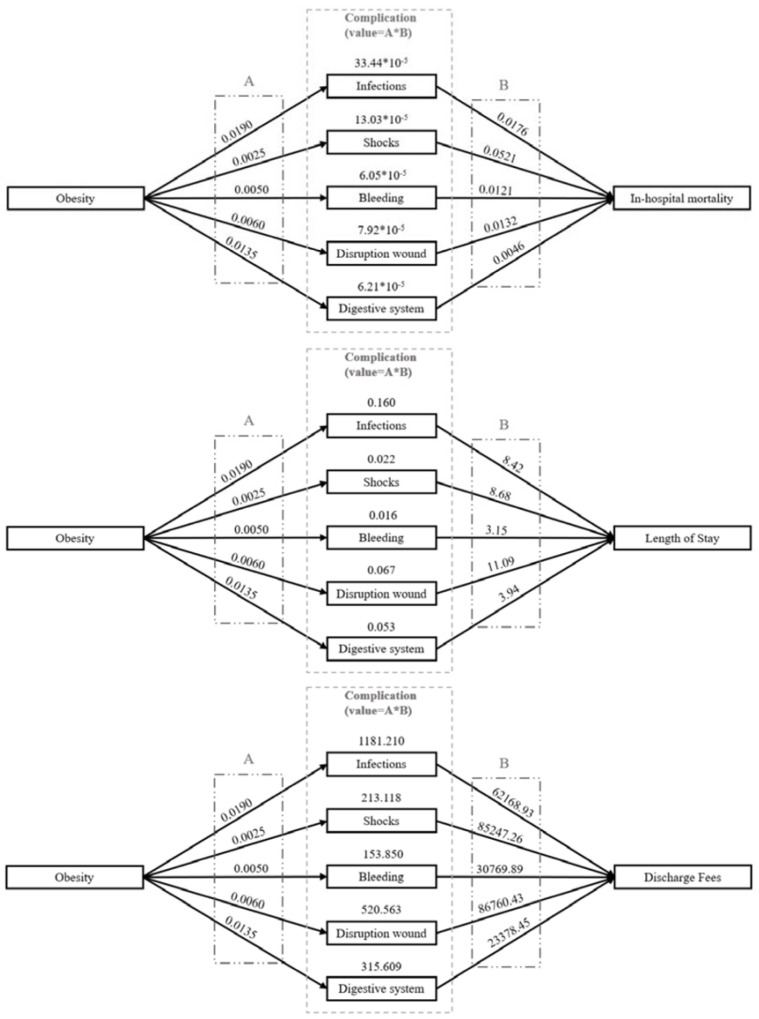
The pathways by which obesity affects outcomes via the complications that were identified as significant.

The marginal effects of obesity on outcomes are shown in Figure 2. Obesity increased in-hospital mortality by 66.65 × 10^−5^%, length of hospital stay by 0.32 days, and total hospital charges by 2384 US dollars. Obesity increases the risk of these outcomes mainly by triggering the complication of infection (33.44 × 10^−5^%, 0.160 days, and 1181 US dollars, respectively).

## 4. Discussion

The purpose of this study is to use a US nationwide database of hospital admissions to examine the effect of obesity on the outcomes of patients with CRC who undergo surgery. The results showed that obesity was associated with an increased risk of postoperative infection, shock, postoperative bleeding, wound disruption, and digestive system complications. Path analysis showed that obesity increased the in-hospital mortality rate through these five postoperative complications, increased the length of hospital stay, and increase the total hospital charges.

In an earlier study, Hussan et al. [9] also used data from the 2012 NIS to study the effects of morbid obesity on the outcomes of patients with CRC who received surgery. In their cohort, approximately 5% of the patients were morbidly obese (defined as a body mass index [BMI] ≥ 40 kg/m^2^). Morbid obesity was associated with an increased risk of peri-operative mortality (odds ratio [OR] = 1.79), an increased length of hospitalization (1.22 days), and an increase in total hospital charges of approximately $15,000.

Obesity was founded to be associated with several postoperative complications. This finding is not unexpected and is consistent with the results of other studies. For example, Tjeertes et al. [8] analyzed the postoperative outcomes of more than 4000 patients receiving general surgical procedures at a single-center. They found that obesity was associated with significantly greater surgical blood loss, longer operation time, and wound infections. A recent meta-analysis found that obese patients who receive surgery for CRC are more likely to have a longer surgery duration, a higher failure rate of minimally invasive approaches, and more likely to have lymph node metastasis [11]. A study of the Swedish National Quality Registry found that longer operating times and greater perioperative bleeding occur in obese patients undergoing surgery for CRC, and these are contributing factors to a higher postoperative complication rate in these patients [12]. Studies have suggested that obesity-induced immune system dysregulation, or obesity-induced reduction in cell-mediated immune response may be the possible mechanisms [13,14,15].

While the results indicated that obesity increased the in-hospital mortality rate of CRC patients receiving surgery, Maskarinec et al. [4] reported that excess body weight had little effect of CRC-specific survival. The authors concluded that while obesity may be an etiological factor for CRC, it has little effect on survival. Another population-based study in the Netherlands indicated that obesity-related comorbidities were associated with a greater postoperative morbidity rate, increased length of hospitalization, and a higher readmission rate in obese patients undergoing CRC surgery [16]. On the other hand, being obese was associated with improved long-term survival, a finding that has been reported in other studies and termed the “obesity paradox”.

A prior review of the literatures to determine the effects of obesity and obesity-related conditions on the prognosis of patients with CRC indicated that metabolic conditions related to obesity (e.g., diabetes and metabolic syndrome), and systemic inflammation related to obesity contribute to the prognosis of patients with CRC [10]. Although it is clear that obesity increases the risk of developing CRC, the exact underlying mechanism is not clear. Inflammation, metabolic syndrome, insulin resistance, changes in levels of adipocytokines, gut microbiota, and bile acids have all been implicated [2,17]. The findings of the present study indicate that obesity increases the risk of these outcomes mainly by triggering the complication of infection. In other words, the monitoring and control of postoperative infection in obese patients is a priority and important task.

### Strengths and Limitations

There are both strengths and limitations to the current study. An important strength of the study is that it used data from a large and high-quality database of hospital care in the United State. The NIS is the largest publicly available all-payer inpatient health care database in the United States, and when weighted contains data of more than 35 million hospitalizations annually cover the whole United States. As such, in addition to providing high-quality data, discrepancies, and biases are minimized. The innovation of this study lays in (1) establishing a structured statistical framework for analysis based on the clinical path model was rarely seen in previous medical researches. (2) Marginal effect of the absolute probability of the presented analysis method could provide more precise interpretations on the association between obesity and CRC than conventionally relative probability did. They are the modeling with path analysis and the concept of absolute marginal effect. In multiple regression, each predictor variable has a direct effect on the response variable. What if the predictor variable (obesity in this study) affects the response variable directly and indirectly through one or more intervening variables (complications in this study). Path analysis is a technique for analyzing this causal relationship [18] and is therefore applied in this study. It builds on ordinary multiple regression and other counterparts for each path. As specified by the clinical path diagram in this study, the causal ordering is derived from theory rather than data themselves.

There are, however, limitations to the study as well. First, this is a retrospective study; the credibility is weaker than RCT or prospective studies. The study relied on ICD-9-CM codes, and the accuracy of the ICD-9-CM coding could not be verified. In addition, only patients with significant pathologic obesity were attributed the relative ICD-9-CM code, which means many patients in the non-obese group might be obese. However, this means that when comparing obese with actual non-obese patients, the results should be more significant. Differences in the degree of obesity were not examined in this study. As the data are de-identified, longitudinal follow-up after discharge cannot be performed. The database does not include services provided in physician offices and does not include complete or reliable pharmacy, laboratory, pathology, or radiology information, all of which may be confounding factors. Limited by the database, this study only explored the impact of obesity. As for the crucial factors causing obesity, such as diet, lifestyle, activity, etc., none was included in the study. Last, the study design included many different surgical procedures related to CRC, including open and minimally invasive techniques. Different surgical approaches themselves may bring differences in postoperative outcomes. It is recommended to have follow-up studies targeting a specific operation or to compare whether differences in outcomes exist between these surgical approaches.

## 5. Conclusions

Obesity increases the risk of postoperative complications in patients with CRC undergoing surgery. Path analysis showed that obesity increased in-hospital mortality through five complications by 66.65 × 10^−5^%, length of hospital stays by 0.32 days, and total hospital charges by 2384 US dollars. Patients with CRC who are obese may require a higher level of preoperative interventions such as weight reduction, blood sugar control, or postoperative complications monitoring to improve outcomes.

## Figures and Tables

**Table 1:** Patient characteristic before and after 1:4 matching.

	Unmatched			Matched		
	Non-Obese *n* = 98,655	Obese *n* = 8412	*p*	Non-Obese *n* = 33,644	Obese *n* = 8411	*p*
**Demography**						
**Sex**						
Male						
Female	49,223 (49.9%)	4468 (53.12%)	<0.0001	17,872 (53.12%)	4468 (53.12%)	1
**Age (years)**						
20–44	4800 (4.87%)	438 (5.21%)	<0.0001	1752 (5.21%)	438 (5.21%)	1
45–59y	21,048 (21.33%)	2479 (29.47%)		9916 (29.47%)	2479 (29.47%)	
60–74y	34,979 (35.46%)	3779 (44.92%)		15,116 (44.93%)	3779 (44.93%)	
75+	37,828 (38.34%)	1716 (20.4%)		6860 (20.39%)	1715 (20.39%)	
**Race**						
White	76,725 (77.77%)	6448 (76.65%)	<0.0001	25,745 (76.52%)	6448 (76.66%)	<0.0001
Black	9634 (9.77%)	1039 (12.35%)		3379 (10.04%)	1039 (12.35%)	
Hispanic	6460 (6.55%)	601 (7.14%)		2313 (6.87%)	601 (7.15%)	
Asian or Pacific Islander	3018 (3.06%)	88 (1.05%)		1173 (3.49%)	88 (1.05%)	
Other	2818 (2.86%)	236 (2.81%)		1034 (3.07%)	235 (2.79%)	
**Household income**						
Q1	24,532 (24.87%)	2225 (26.45%)	<0.0001	8052 (23.93%)	2224 (26.44%)	<0.0001
Q2	24,934 (25.27%)	2124 (25.25%)		8439 (25.08%)	2124 (25.25%)	
Q3	24,257 (24.59%)	2155 (25.62%)		8346 (24.81%)	2155 (25.62%)	
Q4	24,932 (25.27%)	1908 (22.68%)		8807 (26.18%)	1908 (22.68%)	
**Elective admission**	64,127 (65%)	5935 (70.55%)	<0.0001	22,779 (67.71%)	5934 (70.55%)	<0.0001
CRC severity/comorbidities						
Severity of CRC	34,884 (35.36%)	2581 (30.68%)	<0.0001	10,320 (30.67%)	2580 (30.67%)	1
Alcohol abuse	1896 (1.92%)	145 (1.72%)	0.2021	655 (1.95%)	145 (1.72%)	0.1807
Diabetes	18,473 (18.72%)	3425 (40.72%)	<0.0001	5877 (17.47%)	3425 (40.72%)	<0.0001
Liver disease	1809 (1.83%)	249 (2.96%)	<0.0001	633 (1.88%)	249 (2.96%)	<0.0001
Hospital						
**Bed size**						
Small	12,136 (12.3%)	944 (11.22%)	0.009	4024 (11.96%)	943 (11.21%)	0.1192
Medium	24,109 (24.44%)	2121 (25.21%)		8276 (24.6%)	2121 (25.22%)	
Large	62,410 (63.26%)	5347 (63.56%)		21,344 (63.44%)	5347 (63.57%)	
**Location**						
Rural	11,134 (11.29%)	800 (9.51%)	<0.0001	3581 (10.64%)	800 (9.51%)	0.0024
Urban	87,521 (88.71%)	7612 (90.49%)		30,063 (89.36%)	7611 (90.49%)	
**Teaching hospital**	43,760 (44.36%)	3937 (46.8%)	<0.0001	14,904 (44.3%)	3937 (46.81%)	<0.0001

CRC, colorectal cancer. Bold indicates significant difference between obese and non-obese groups, *p* < 0.05.

**Table 2:** Outcomes before and after 1:4 matching.

	Unmatched			Matched		
	Non-Obese	Obese	*p*	Non-Obese	Obese	*p*
**Outcome**						
In-hospital mortality	2977 (3.02%)	148 (1.76%)	<0.0001	764 (2.27%)	148 (1.76%)	0.0039
Postoperative infection	4402 (4.46%)	526 (6.25%)	<0.0001	1419 (4.22%)	526 (6.25%)	<0.0001
Postoperative shock	398 (0.4%)	48 (0.57%)	0.0223	99 (0.29%)	48 (0.57%)	0.0001
Postoperative bleeding	2223 (2.25%)	214 (2.54%)	0.0862	672 (2%)	214 (2.54%)	0.0018
Wound disruption	1281 (1.3%)	147 (1.75%)	0.0006	358 (1.06%)	147 (1.75%)	<0.0001
Digestive system complications	12,395 (12.56%)	1106 (13.15%)	0.1215	3957 (11.76%)	1106 (13.15%)	0.0005
LOS ^a^ (days)						
Mean ± SD	9.68 ± 8.17	9.52 ± 7.93	0.0641	9.08 ± 8.02	9.52 ± 7.93	<0.0001
Median	7	7		7	7	
Total hospital charges (US dollars) ^b^						
Mean ± SD	68,547 ± 78,220	74,541 ± 82,478	<0.0001	59,463 ± 68,653	74,545 ± 82,483	<0.0001
Median	46,456	51,745		40,420	51,762	
**Missing data**						
In-hospital mortality	61	0		9	0	
LOS (days)	2	0		1	0	
Total hospital charges (US dollars)	2651	503		1131	503	

^a^: LOS, length of hospital stay; SD, standard deviation. ^b^: Round to whole number. Bold indicates significant difference between obese and non-obese groups, *p* < 0.05.

**Table 3:** Logistic regression analysis of the effect of obesity on colorectal cancer postoperative complications †.

	Infection			Shock			Bleeding		
Explanatory Variables	ß	OR	*∂*P/*∂*X	ß	OR	*∂*P/*∂*X	ß	OR	*∂*P/*∂*X
**Obesity **	0.43	1.54 (1.39, 1.71)	0.0190	0.72	2.05 (1.45, 2.91)	0.0025	0.24	1.28 (1.09, 1.49)	0.0050
**Race**									
Other	−0.02	0.98 (0.75, 1.29)	−0.0008	0.36	1.44 (0.63, 3.28)	0.0013	0.15	1.16 (0.8, 1.67)	0.0030
Asian or Pacific Islander	0.23	1.26 (0.99, 1.62)	0.0103	0.64	1.9 (0.92, 3.92)	0.0022	−0.16	0.86 (0.56, 1.32)	−0.0032
Hispanic	0.004	1 (0.84, 1.2)	0.0002	0.17	1.19 (0.65, 2.18)	0.0006	0.02	1.02 (0.78, 1.33)	0.0004
Black	0.02	1.02 (0.88, 1.18)	0.0009	−0.12	0.89 (0.49, 1.59)	−0.0004	−0.04	0.96 (0.76, 1.21)	−0.0009
**Household income**									
Q4	0.06	1.06 (0.93, 1.22)	0.0027	0.59	1.81 (1.09, 3.01)	0.0021	−0.02	0.98 (0.8, 1.2)	−0.0004
Q3	0.05	1.05 (0.92, 1.2)	0.0022	0.48	1.61 (0.98, 2.67)	0.0017	0.11	1.11 (0.92, 1.36)	0.0022
Q2	0.01	1.01 (0.88, 1.15)	0.0004	0.30	1.36 (0.81, 2.28)	0.0011	0.09	1.09 (0.9, 1.33)	0.0018
**Elective Admission**	−0.44	0.64 (0.58, 0.71)	−0.0195	−0.65	0.52 (0.38, 0.73)	−0.0022	−0.06	0.94 (0.81, 1.09)	−0.0013
**Hospital location**									
Urban	0.04	1.05 (0.88, 1.25)	0.0020	1.06	2.87 (1.15, 7.17)	0.0037	−0.05	0.96 (0.75, 1.21)	−0.0009
**Teaching Hospital**	0.33	1.39 (1.26, 1.53)	0.0144	−0.10	0.91 (0.65, 1.26)	−0.0003	0.08	1.09 (0.94, 1.25)	0.0017
Constant	−3.08		−0.1359	−6.80		−0.0237	−3.89		−0.0803
*N*	1945			147			886		
Likelihood ratio	190.5245			47.52654			15.02153		
−2 log *L*	15, 564.86			1908.912			8578.129		
Chi−square P	205.1729			42.04551			15.11302		
Dependent variable mean	0.046			0.003			0.021		
	Wound disruption					Digestive system complications			
**Explanatory Variable**	**ß**		**OR**	***∂*P/*∂*X**		ß	**OR**		***∂*P/*∂*X**
**Obesity**	0.51		1.66 (1.37, 2.02)	0.0060		0.13	1.14 (1.06, 1.22)		0.0135
**Race**									
Other	−0.47		0.63 (0.33, 1.18)	−0.0056		−0.10	0.91 (0.76, 1.08)		−0.0104
Asian or Pacific Islander	0.003		1 (0.59, 1.72)	0.00004		−0.10	0.9 (0.75, 1.08)		−0.0110
Hispanic	0.08		1.08 (0.78, 1.5)	0.0009		−0.18	0.84 (0.74, 0.95)		−0.0188
Black	−0.10		0.91 (0.68, 1.21)	−0.0012		0.10	1.1 (1, 1.21)		0.0102
**Household income**									
Q4	−0.28		0.76 (0.58, 0.99)	−0.0033		−0.04	0.96 (0.88, 1.05)		−0.0044
Q3	−0.07		0.94 (0.73, 1.2)	−0.0008		0.01	1.01 (0.93, 1.1)		0.0010
Q2	−0.02		0.98 (0.77, 1.25)	−0.0002		−0.02	0.98 (0.9, 1.07)		−0.0018
**Elective Admission**	−0.61		0.54 (0.45, 0.65)	−0.0072		−0.17	0.85 (0.79, 0.9)		−0.0178
**Hospital location**									
Urban	−0.24		0.79 (0.58, 1.07)	−0.0028		0.15	1.16 (1.04, 1.29)		0.0155
**Teaching Hospital**	0.46		1.58 (1.31, 1.92)	0.0055		−0.09	0.91 (0.86, 0.97)		−0.0096
Constant	−4.07			−0.0483		−1.98			−0.2092
N	505					5063			
Likelihood ratio	94.76488					71.62981			
−2 log *L*	5375.543					30, 855.72			
Chi-square P	98.17657					71.09101			
Dependent variable mean	0.012					0.120			

OR, odds ratio. † *∂*P/*∂*X represents the marginal probability change when independent variable (X) changes one unit, it equals ß × P × (1 − P), where P is the probability of complications occurred.

**Table 4:** Logistic regression analysis of the effect of complications on in-hospital mortality †.

	In-Hospital Mortality		
Explanatory Variables	ß	OR	*∂*P/*∂*X
Infection	0.83	2.3 (1.82, 2.9)	0.0176
Shock	2.45	11.64 (7.66, 17.68)	0.0521
Bleeding	0.57	1.77 (1.27, 2.47)	0.0121
Wound disruption	0.62	1.86 (1.23, 2.81)	0.0132
Digestive system complications	0.22	1.24 (1.03, 1.5)	0.0046
Constant	−3.97		−0.0842
*N*	912		
Likelihood ratio	218.2210		
−2 log *L*	8573.377		
Chi-square P	306.0189		
Dependent variable mean	0.022		

OR, odds ratio. † *∂*P/*∂*X represents the marginal probability change when independent variable (X) changes one unit, it equals ß × P × (1 − P), where P is the probability of in-hospital mortality.

**Table 5:** Ordinary least squares regression examining the effect of complications on length of hospital stay and total hospital charges.

	Length of Stay		Total Charges (US Dollars) ^a^	
Explanatory Variables	ß	CI	ß	CI
Infection	8.42	(8.07, 8.77)	62,169	(58,887, 65,451)
Shock	8.68	(7.46, 9.89)	85,247	(73,746, 96,748)
Bleeding	3.15	(2.65, 3.65)	30,770	(26,075, 35,464)
Wound disruption	11.09	(10.43, 11.76)	86,760	(80,563, 92,958)
Digestive system	3.94	(3.72, 4.16)	23,378	(21,302, 25,455)
Constant	8.08	(8, 8.15)	54,795	(54,067, 55,523)
*N*	42,054		40,421	
R^2^	0.1332		0.0886	
Adjusted R^2^	0.1331		0.0885	

^a^: Round to whole number.

## Data Availability

Data supporting generated results are available by the corresponding author.

## Supplementary Materials

The following are available online at https://www.mdpi.com/article/10.3390/jcm10132904/s1, Table S1: ICD-9 codes used in this study.
