# Supplementary material for: Path Analysis of the Impact of Obesity on Postoperative Outcomes in Colorectal Cancer Patients: A Population-Based Study
Source: J Clin Med. 2021 Jun 29;10(13):2904. doi: 10.3390/jcm10132904 (PMC8268380; doi:10.3390/jcm10132904)
Supplement: Supplementary file 1 [file jcm-10-02904-s001.zip › jcm-1207341-supplementary.pdf]

Supplementary Table S1. ICD-9 codes used in this study.

Term	ICD 9 CM/Procedure
Colorectal cancer	153, 154
Excluded diagnosed with other cancer -	140-239
Surgical intervention	
Open and subtotal colectomy	45.7
pull-through resection of the rectum	48.40, 48.41, 48.43, 48.49
abdominoperineal resection of the rectum/complete proctectomy	48.50, 48.52, 48.59
other resections of the rectum/partial proctectomy /rectosigmoidectomy	48.6
Primary endpoint	
postoperative infection	998.5
postoperative shock	998.0
postoperative bleeding	998.1
disruption of wound	998.3
non-healing surgical wound	998.83
nervous system complications	997.0
cardiac arrest/heart failure	997.1
phlebitis/thrombophlebitis	997.2
respiratory complications	997.3
digestive system complications	997.4
urinary complications	997.5
vascular complications	997.7
unspecified complications	998.9
